# Supplementary material for: The causal relationship between the human gut microbiota and pyogenic arthritis: a Mendelian randomization study
Source: Front Cell Infect Microbiol. 2024 Nov 26;14:1452480. doi: 10.3389/fcimb.2024.1452480 (PMC11629706; doi:10.3389/fcimb.2024.1452480)
Supplement: Supplementary file 1 [file DataSheet1.zip › mendelian test/ebi-a-GCST90027492.csv_forest.pdf]

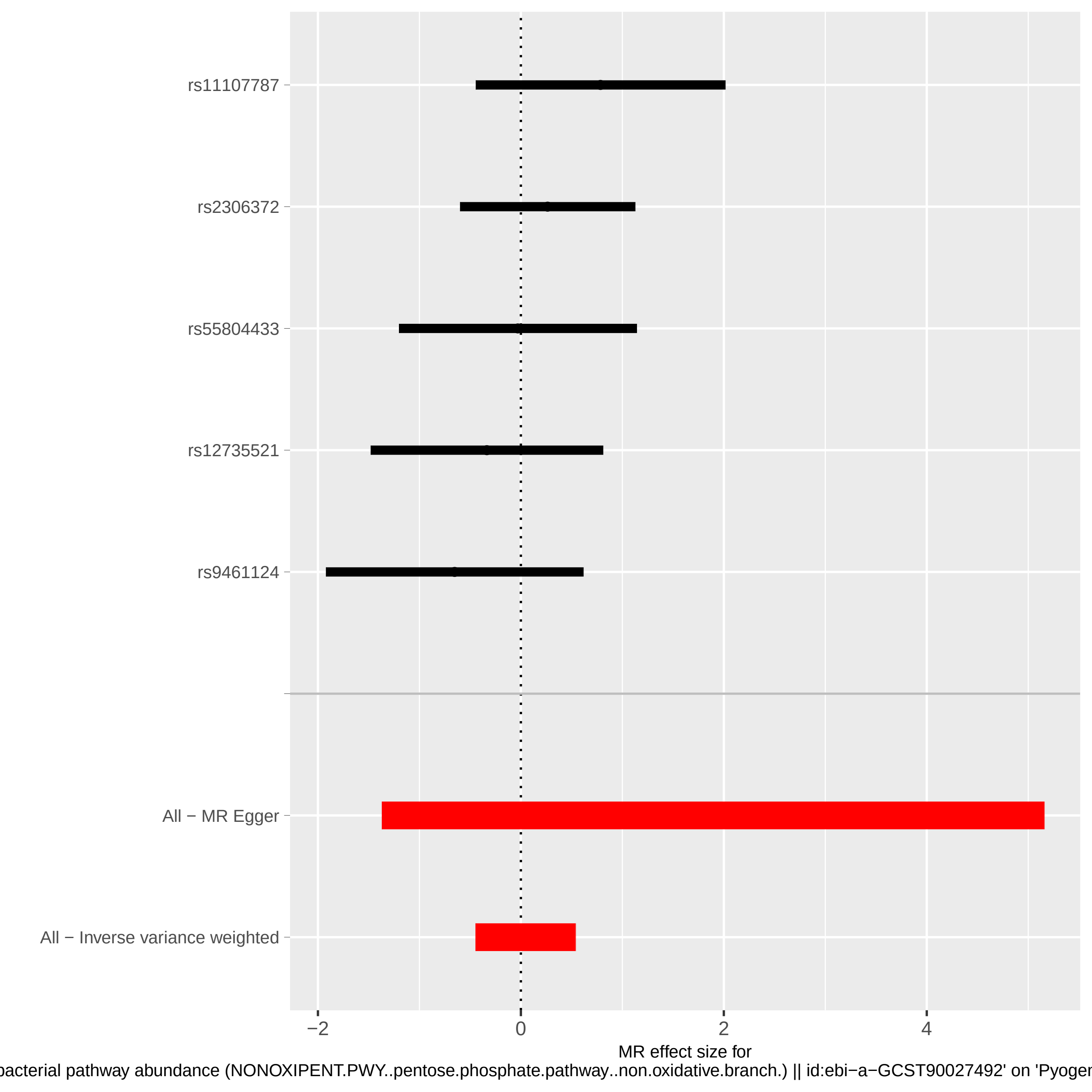
rs11107787
rs2306372
rs55804433
rs12735521
rs9461124
All − MR Egger
All − Inverse variance weighted
−2
0
2
4
MR effect size for
bacterial pathway abundance (NONOXIPENT.PWY..pentose.phosphate.pathway..non.oxidative.branch.) || id:ebi−a−GCST90027492' on 'Pyoger
